# Improving Language Acquisition and Processing With Cognitive Stimulation

**DOI:** 10.3389/fpsyg.2021.663773

**Published:** 2021-05-14

**Authors:** José Luis Tapia, Jon Andoni Duñabeitia

**Affiliations:** ^1^Centro de Ciencia Cognitiva (C3), Universidad Antonio de Nebrija, Madrid, Spain; ^2^AcqVA Aurora Center, The Arctic University of Norway, Tromsø, Norway

**Keywords:** cognitive stimulation, brain training, language learning, executive functions, intervention programs

## Introduction

Cognitive functions are essential in human development in general, and they play a key role in language learning, as well as in reading and writing. A large body of evidence makes the relationship between executive functions and language acquisition and processing indisputable [Moser et al., 2007; Mazuka et al., 2009; Woodard et al., 2016; see also the meta-analysis by Swanson et al. (2009)]. Lexical-semantic processing has been associated with inhibition skills (Khanna and Boland, 2010) and with working memory and information updating (Weiland et al., 2014), whereas syntactic processing has been linked with inhibition, shifting, updating (Novick et al., 2005; Roberts et al., 2007). Memory updating has been suggested to underlie both sentence comprehension (Daneman and Carpenter, 1980) and production (Slevc, 2011). Furthermore, executive functions have also been correlated with the development of phonological awareness (Risso et al., 2015). Broadly speaking, the neuroscientific literature has consistently shown that executive functions and language skills are interrelated, suggesting an overlap of the neural processes involved [see Slot and Von Suchodoletz (2018)].

Despite the large body of research demonstrating the close link between executive functions and language skills, it is yet to be established the possible bidirectionality or reciprocality between the development of both macro-systems and the associated skills. Following the notion of brain plasticity and the expansion-partial renormalization hypothesis (EPH) (Pliatsikas, 2020), any new cognitive effort, such as acquiring a new language, may produce a change in the neural system and pathways (e.g., increasing the number of synapses, generating new dendritic spines, or strengthening neural connections) related to this learned skill. With this being so, and using digital biomarkers, one could potentially quantify the specific changes in the cognitive system induced by language learning, but more importantly, one could also determine the best cognitive foundations on which language learning could be built by virtue of establishing the reciprocal connections between domain-general executive functions and language acquisition. Furthermore, as Rojas-Barahona et al. (2015) proposed, a cognitive stimulation intervention focused on these biomarkers could potentially increase and strengthen the neural network underlying language skills.

## Cognitive Stimulation And Brain Training: Concept And Effectiveness

Although cognitive stimulation, cognitive training, and brain training may represent interchangeable terms, a correct nomenclature should be adopted to correctly narrow down the extent to which a given intervention is expected to impact cognitive behavior. Cognitive stimulation typically refers to all those techniques and strategies that aim to improve the cognitive functioning of different capacities and cognitive functions such as attention, reasoning, memory, perception, abstraction, language, or praxis. Cognitive stimulation seems to be more appropriate than the other terms, since these interventions do not really “train” the brain as an organ but stimulate its activity and the cognitive functions. Hence, cognitive stimulation stands as an all-encompassing umbrella term that could adequately account for the different intervention protocols aimed to improve cognitive functions and/or the underlying neural bases and mechanisms.

In the 1970s, clinical intervention programs were designed aimed at the restoration of damaged cognitive functions focusing mainly on the cognitive domains of attention, executive functions, working memory, processing speed, and reasoning. A large number of therapists began to use cognitive training as a path to neuropsychological rehabilitation of patients with brain injury (Sohlberg and Mateer, 1987), depression (Zeiss et al., 1979), cognitive impairment (Labouvie-Vief and Gonda, 1976), hyperactivity (Douglas et al., 1976), or schizophrenia (Olbrich and Mussgay, 1990). These protocols soon began to be criticized given the presence of methodological shortcomings. As Abikoff (1979) reviewed, in most cases, interventions for cognitive rehabilitation resulted in positive outcomes in the directly trained functions, with none or limited transferability to other cognitive skills or everyday functioning [see also Wilson (1997)].

Early in the twenty-first century, the market around commercial cognitive stimulation programs started to become increasingly prominent, reigniting the interest in the field. Since 1999, when a group of scientists and academics created a company to promote cognitive training and launched CogniFit (CogniFit Inc., San Francisco, US), many other companies have created similar products with different degrees of success (e.g., Cogmed, now property of Pearson Education, London, UK; BrainHQ by Posit Science Corporation, San Francisco, US). In 2005 the Japanese market revolutionized the field when Brain Age (Nintendo Co. Ltd., Kyoto, Japan) was first launched, and it was soon extended to the American and European market in 2006. Due to the rise and spread of these programs, the US Federal Trade Commission started to arbitrate the market, sueing some companies for deceptive advertising, and giving rise to an agitated period in which the effectiveness of cognitive stimulation programs was questioned [see Allaire et al. (2014), for a letter claiming for inflated and misleading results because of a long-standing industry behind these products; but see Alescio-Lautier et al. (2014), for a response letter in defense of the efficacy of cognitive stimulation programs].

Over the years, cognitive stimulation has been used in a wide variety of areas, such as learning and education (Melby-Lervåg and Hulme, 2013), psychological disorders (Lawlor-Savage and Goghari, 2014), brain damage (Spreij et al., 2014) or neurodegenerative disorders (Reijnders et al., 2013), reporting improvements in overall cognition and in specific cognitive domains in healthy and unhealthy samples [see Wang et al. (2016)]. In this vein, cognitive stimulation programs have adopted multiple forms and approaches, ranging from scientific interventions based on neuroplasticity (Nahum et al., 2013) and cognitive constructs (Jaeggi et al., 2008) or approaches inspired by meditation practices (Tang et al., 2007), to more gamified interventions (Anguera et al., 2013), or to commercial videogames originally designed for other purposes (Green and Bavelier, 2003). This diversity of target populations and approaches has been partially responsible for the controversy on the reproducibility and generalizability of the results obtained across studies and on the effectiveness of cognitive stimulation programs [see Fisher (2014)].

The effectiveness of an intervention based on a cognitive stimulation program could be measured from two viewpoints. On the one hand, a cognitive stimulation program could be considered effective as long as the specific function worked significantly improves between a pre-intervention evaluation and a post-intervention assessment. On the other hand, the effectiveness of a cognitive stimulation program could be also understood as the improvements found in general cognitive functioning -far effects- and not only in the trained domain -near effects- (Lindenberger et al., 2017). According to this last view, for an intervention to be effective, improvements should be also found in untrained tasks and daily functioning (Simons et al., 2016). Most of the evidence obtained in cognitive stimulation interventions has focused on improvements in the tasks or domains directly worked (i.e., near transfer effects; see van Heugten et al., 2016, for review), but far transfer effects have been also reported, although to a lesser degree [see Dahlin et al. (2008); Hardy et al. (2015); see Au et al. (2015), for a meta-analysis].

## Optimization Of Cognitive Stimulation Programs

The most relevant quest to tackle in cognitive stimulation research is precisely how to achieve far transfer effects. Here we propose three different strategies that could be combined to foster generalization to other untrained mechanisms or skills: the ecological validity and capacity of adherence of the intervention, the length and spacing of the training and its sessions, and the dynamic adaptation of the program to the cognitive state of each individual at each stage of the process.

First, we would like to stress out the importance of designing ecologically relevant and motivating protocols. Training a specific skill or even a specific cognitive domain will presumably bring off improvements in those areas. However, in order to find an impact on daily operations and functioning, intervention programs would necessarily contemplate everyday life problems and life-like tasks, including social interactions (Engert et al., 2017; Valk et al., 2017). Besides, motivation may function as a hidden factor masking far transfer effects and modulating adherence and dropout rates. Participants' expectations about a given intervention's outcome and their subsequent motivation toward it can drastically change the observed effects (Finniss et al., 2010; Rutherford et al., 2010; Boot et al., 2013; Keitel et al., 2013).

Second, the establishment of a scientifically validated training timespan needed to obtain far transfer effects is needed. While in some studies participants train for a short period of time every day, in others they train only some days a week, showing great variability in the training time from one study to another. Besides, the structure of some cognitive stimulation programs lasts for weeks (e.g., Knapp et al., 2006), while others can last for years.

And third, the natural variability associated with baseline performance across the set of the cognitive domains or abilities that will be trained is a critical factor that could modulate the effectiveness of any intervention. In other words, the inter-individual differences of two trainees with different baseline levels of cognitive skills and different improvement paces would require that cognitive stimulation programs adjust the level of complexity or difficulty of the set of tasks to offer tailor-made interventions. While some programs present seemingly scalar levels of difficulty that statically adapt to certain initial user profiles, the key aspect is the inclusion of fully adaptive algorithms that adjust the difficulty levels and intensity of the training dynamically throughout the process. Hence, the dynamic adaptation of any cognitive stimulation program to the cognitive baseline of each trainee stands as an essential requisite to foster not only maximization of the benefits of the training, but also adherence to it.

## Discussion

New technologies make it possible to computerize cognitive stimulation programs in a likable gaming environment. The computerization of the tasks and the development of adaptive difficulty algorithms allow the design of challenging activities that require dynamically changing levels of cognitive effort, yielding an enhancement of the outcomes. This, together with the possibility of adjusting the protocols thanks to the large amount of data collected through the same intervention program across persons and devices, increases the chances to develop data-informed ecologically valid adaptative cognitive stimulation programs and platforms (see the products of CogniFit, as a paradigmatic example).

The brain changes and cognitive adaptation that occur when a person acquires a new language suggest that language could be considered as one of the most natural brain training programs for enhancing cognitive functions [see Luk et al. (2020); Pliatsikas (2020), for review]. Not surprisingly, in the last decade a whole line of research has focused on the impact of multilingualism or of the acquisition of a non-native language in domain-general cognitive skills [see Antón et al. (2019); Leivada et al. (2021)]. But over and above the possibility of exploring and measuring the cognitive milestones attained in the process of language training, it is worth considering also a different approach resulting from taking the opposite view angle. Given the close link between domain-general sets of cognitive skills such as executive functions and language learning, one could tentatively predict that interventions based on cognitive stimulation programs could give rise to enhanced language learning. Put differently, given that learning a second or foreign language could result in far transfer effects in non-linguistic cognitive skills, it could also be expected that specific non-linguistic cognitive training could improve not only the cognitive skills that underlie language control [see Liu et al. (2016, 2019)], but also boost language learning and linguistic skills. While this is a very incipient line of research that is still in its infancy, a handful of studies already bring hope to this approach (e.g., Hayashi, 2019; Karousou and Nerantzaki, 2020).

Interventions based on cognitive stimulation programs have been carried out effectively in all age ranges, both in persons with different pathologies or cognitive dysfunctions and in healthy individuals. With this in mind and taking into account that the largest portion of the world speaks two languages or is learning a non-native language, the inclusion of cognitive stimulation programs in the daily agenda of current and future multilinguals seems a logical step.

## Author Contributions

All authors listed have made a substantial, direct and intellectual contribution to the work, and approved it for publication.

## Conflict of Interest

The authors declare that the research was conducted in the absence of any commercial or financial relationships that could be construed as a potential conflict of interest.
